# Dr. Lanston Parker on the Use of Mercury in the Treatment of Primary Syphilis

**Published:** 1862-03

**Authors:** 


Dr. Lanston Parker on the use of Mercury in the Treatment of Primary Syphilis.—The principal facts inculcated by the author are embodied in the subjoined *resume:*

1—That mercury is not indicated as a specific agent in the treatment of the soft or simple chancre which secretes pus freely, which has no induration of its base, and no complication in the groin.

2—That mercury is not indicated as a specific therapeutic agent in the treatment of the adenitis which complicates this form of chancre, whether the tumor be of a benign or virulent character.

3—That mercury is not indicated in the treatment of the ingunial chancre which succeeds to the opening or the bursting of a virulent bubo.

4—That mercury may be tried when this form of chancre, or the attendant inguinal chancre, is perfectly chronic, and has resisted the usual routine of non-mercurial treatment. In the earlier stages of these diseases mercury is positively injurious; but in the perfectly chronic stage, as a *dernier resort*, it may be tried, and occasionally, but not certainly, its use is attended with benefit. In such states a moist mercurial fumigation is sometimes efficacious—a fact which Mr. Parker has ascertained in several instances.

5—Mercury is occasionally useful in the treatment of the phlegmoid induration which sometimes accompanies soft chancres; here its action is limited to the removal of the induration; it has no influence in dispersing either of the forms of adenitis which may complicate it.

6—Mercury is indicated as a specific agent in the treatment of the infecting or specifically indurated chancre. It is the most powerful and certain therapeutic that can be opposed to it. It resolves the indolent buboes which almost always accompany this chancre, and which remain stationary or continue to increase until mercury is used. It weakens or altogether eradicates the dyscrasia, or constitution taint, of which this chancre is the initiatory symptom.

7—Mercury may be given internally, and by way of friction, or administered in the form of vapor. There are cases in which each of these modes may find its special application. Generally speaking, the internal administration is the most injurious to the patient and the least efficacious in its influence on the disease.—*Medical Times and Gazette—Reporter.*

